# MIF1 and MIF2 Myostatin Peptide Inhibitors as Potent Muscle Mass Regulators

**DOI:** 10.3390/ijms23084222

**Published:** 2022-04-11

**Authors:** Eun Ju Lee, Sibhghatulla Shaikh, Mohammad Hassan Baig, So-Young Park, Jeong Ho Lim, Syed Sayeed Ahmad, Shahid Ali, Khurshid Ahmad, Inho Choi

**Affiliations:** 1 Department of Medical Biotechnology, Yeungnam University, Gyeongsan 38541, Korea; gorapadoc0315@hanmail.net (E.J.L.); sibhghat.88@gmail.com (S.S.); lim2249@naver.com (J.H.L.); sayeedahmad4@gmail.com (S.S.A.); ali.ali.md111@gmail.com (S.A.); 2 Research Institute of Cell Culture, Yeungnam University, Gyeongsan 38541, Korea; 3 Department of Family Medicine, Gangnam Severance Hospital, Yonsei University College of Medicine, Seoul 06273, Korea; mohdhassanbaig@gmail.com; 4 Department of Physiology, College of Medicine, Yeungnam University, Daegu 42415, Korea; sypark@med.yu.ac.kr

**Keywords:** skeletal muscle, peptides, myostatin, myogenesis, muscle regeneration

## Abstract

The use of peptides as drugs has progressed over time and continues to evolve as treatment paradigms change and new drugs are developed. Myostatin (MSTN) inhibition therapy has shown great promise for the treatment of muscle wasting diseases. Here, we report the MSTN-derived novel peptides MIF1 (10-mer) and MIF2 (10-mer) not only enhance myogenesis by inhibiting MSTN and inducing myogenic-related markers but also reduce adipogenic proliferation and differentiation by suppressing the expression of adipogenic markers. MIF1 and MIF2 were designed based on in silico interaction studies between MSTN and its receptor, activin type IIB receptor (ACVRIIB), and fibromodulin (FMOD). Of the different modifications of MIF1 and MIF2 examined, _Ac_-MIF1 and _Ac_-MIF2-_NH2_ significantly enhanced cell proliferation and differentiation as compared with non-modified peptides. Mice pretreated with _Ac_-MIF1 or _Ac_-MIF2-_NH2_ prior to cardiotoxin-induced muscle injury showed more muscle regeneration than non-pretreated controls, which was attributed to the induction of myogenic genes and reduced MSTN expression. These findings imply that _Ac_-MIF1 and _Ac_-MIF2-_NH2_ might be valuable therapeutic agents for the treatment of muscle-related diseases.

## 1. Introduction

Skeletal muscle (SM) comprises ~40% of body weight and is the most dynamic organ, with a tremendous ability to regenerate and repair after injury or trauma [1,2]. SM is responsible for the maintenance of postural support, movement, thermogenesis, and blood glucose homeostasis [3,4]. Protein synthesis and degradation homeostasis are required for the maintenance of healthy muscle mass and for sensitivity to physical activity, hormonal balance, injury, and nutritional status [1]. The IGF1-Akt-mTOR pathway is a positive regulator of protein synthesis and is counterbalanced by the myostatin—Smad2/3 pathway, which acts as a negative regulator [5]. Imbalance in the myostatin—Smad2/3 pathway causes muscle atrophy, which, in the context of cancer, is called cancer cachexia [6]. Naturally, muscle loss occurs from 35 years of age and ~30% loss occurs between the ages of 50 and 80 [7]. SM loss is the main characteristic of aging and ailments such as obesity, diabetes, and cancer [8,9].

SM contains a diverse population of muscle satellite cells (MSCs), which are multipotent precursor cells present between the sarcolemma and basal lamina that provide anatomical and functional stability, thus preserving SM integrity [10,11,12]. MSCs have self-renewing ability and can differentiate to form myotubes via a myogenic program that is dependent on the coordinated actions of paired box transcription factors (Pax3/Pax7) and the basic helix-loop-helix family of transcription factors (myogenic factor 5, Myf5; myogenin, MYOG; and myogenic determination factor, MYOD). During the gradual muscle deterioration associated with muscular dystrophies and aging, MSC activity is commonly impaired due to asymmetrical division or abnormal transcriptional control [13].

Myostatin (MSTN), which is referred to as a myokine, belongs to the transforming growth factor β (TGF-β) superfamily and, as mentioned above, acts as a negative regulator of muscle growth. Serum MSTN has been reported to be a major risk factor of pre-sarcopenia and sarcopenia [14,15], whereas MSTN knockout (MSTN^−/−^) mice exhibited greater myofiber size, muscle weight, and grip strength than wild-type controls [16]. Fibromodulin (FMOD) is an extracellular matrix (ECM) gene expressed abundantly in muscle and connective tissues such as cartilage, skin, and tendons [17] and an MSTN regulator that controls muscle cell formation during the myogenic program [18]. Follistatin, another TGF-β superfamily member, has also been reported to be involved in the regulation of muscle size and mass [19,20].

MSTN is expressed in adipose and muscle tissues and plays a vital role during adipogenesis, which it can inhibit or enhance, depending on the situation [21]. Reportedly, MSTN regulates the adipogenesis of mesenchymal stem cells during the differentiation and determination phases [22], and in animals, its deletion or inhibition enhances muscle mass and decreases fat mass [21].

Several strategies have been used to develop treatments for SM-related disorders, and computational approaches provide an impressive means of designing and developing new therapeutics. Computer-aided drug design is widely regarded in the context of drug development [23], and the discovery of promising lead compounds using these approaches provides an effective means of designing compounds with the required therapeutic profiles [24]. Peptides are short-chain amino acids with prodigious characteristics, such as extreme specificity and membrane penetration efficiency, and in addition, they are reasonably inexpensive [25,26]. For these purposes, the design of peptides that mimic specific proteins has enormous therapeutic potential. Peptides have already had major impacts on the pharmaceutical industry and the directions of biological and chemical research [27]. Mimetics are usually designed using the 3D structure of proteins, which are the primary source of active peptides, as peptide fragments that are designed based on known protein–protein interactions (PPIs) are the key factors in rational drug design [28,29]. Therefore, in this study, we aimed to design peptides that promote muscle proliferation and differentiation by targeting MSTN to develop a therapeutic alternative for the treatment of muscle disorders.

## 2. Results

### 2.1. MIF Peptide Design: In Silico Analysis

In-depth analysis of the bindings between MSTN and the activin type IIB receptor (ACVRIIB) or FMOD showed that the residues at positions 22–31 of MSTN were predominantly involved with both interactions. Computational alanine scanning of MSTN–FMOD and MSTN–ACVRIIB complexes showed that MSTN residues in this region were major contributors to ACVRIIB and FMOD binding (Supplementary Tables S1–S3). Changes in accessible surface area (ASA) confirmed the residues in this region of MSTN exhibited maximum ASA changes after binding with FMOD or ACVRIIB.

As can be seen in Supplementary Tables S1–S3, the residues in the 22–31 region of MSTN exhibited large ASA changes after binding to ACVRIIB or FMOD or in complexes with FMOD–ACVRIIB. V22, F24, F27, F27, G28, W29, and W31 residues contributed the most in terms of buried surface area (BSA). Virtual alanine scanning of MSTN/FMOD and MSTN/ACVRIIB showed that V22, F27, W29, and W31 were major contributors in terms of binding free energy when replaced by alanine (Supplementary Tables S4 and S5). Based on these results, the region from 22 to 31 was used to construct inhibitory peptides.

**Figure 1:**
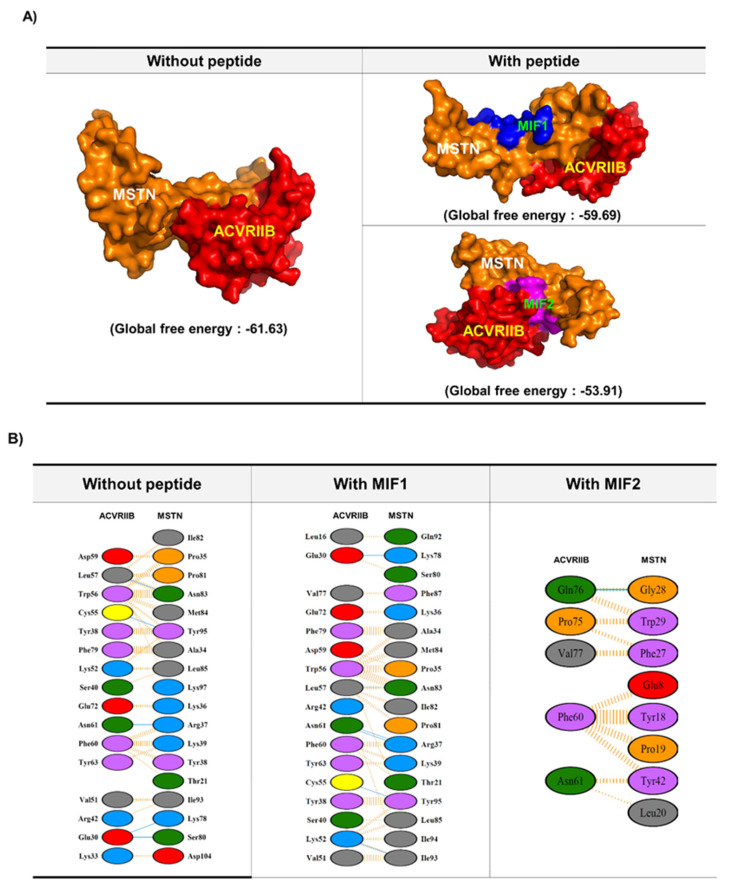
Interactions between MSTN and extracellular domain of ACVRIIB in presence and absence of MIF 1 and 2 peptides. (**A**) Interactions between MSTN and ACVRIIB in the presence and absence of MIF 1 and 2. (**B**) Interaction plot demonstrating the residual interaction between MSTN and the extracellular domain of ACVRIIB in the presence and absence of MIF 1 and 2.

**Figure 2:**
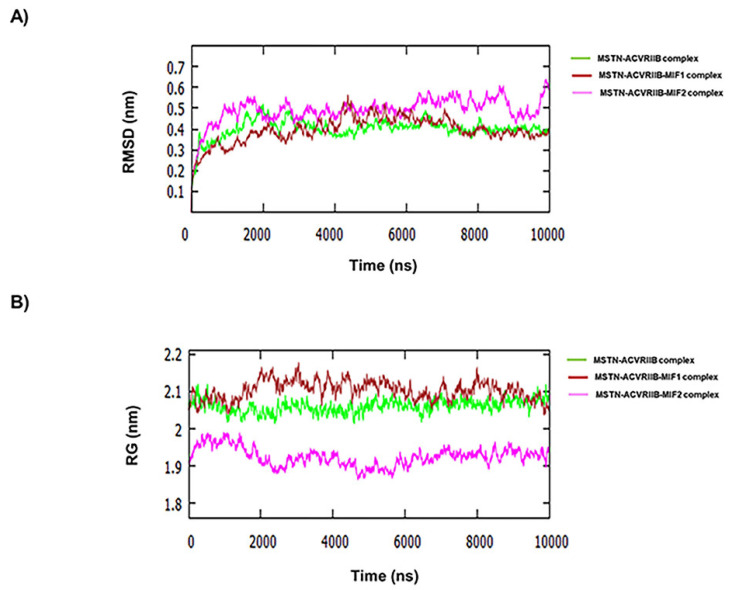
RMSD analysis. (**A**) RMSDs from starting structures for the 10 ns simulations of MSTN–ACVRIIB complex (green), ACVRIIB–MSTN–MIF1 complex (brown), and ACVRIIB–MSTN–MIF2 complex (magenta). (**B**) Changes in radius of gyration (RG) from starting structures for the 10 ns simulations of MSTN–ACVRIIB complex (green), ACVRIIB–MSTN–MIF1 complex (brown), and ACVRIIB–MSTN–MIF2 complex (magenta).

Several peptides comprised of short sequences and modifications of those sequences were generated using these residues. These peptides were screened/docked against MSTN. Furthermore, complexes of MSTN with these peptides were subjected to PPI with ACVRIIB. MIF1 and MIF2 were chosen for further investigation based on global binding scores (Table 1 and Figure 1). As shown in Figure 1A,B, the presence of these peptides hindered the binding between MSTN and ACVRIIB. For instance, MSTN bound to ACVRIIB with a global binding score of −61.63, which was reduced to −59.69 and −53.91 in the presence of MIF1 and MIF2, respectively (Figure 1). The MSTN-ACVRIIB complex was also analyzed for stability by molecular dynamics (MD) simulation in the presence of MIF1 or MIF2. Figure 2A shows the root-mean-square deviation (RMSD) values of the MSTN-ACVRIIB complex in the presence and absence of MIF1 or MIF2 and variations in the conformational flexibilities of MSTN-ACVRIIB, MSTN-ACVRIIB-MIF1, and MSTN-ACVRIIB-MIF2 complexes throughout 10 nanosecond (ns) simulations. The structural flexibility of MSTN-ACVRIIB was found to exhibit fewer structural fluctuations than the peptide-bound models. RMSD fluctuation plots showed that ACVRIIB–MSTN (green) and MIF1-bound ACVRIIB–MSTN (brown) structures were more stable than ACVRIIB-MIF2 (magenta). As shown by the RMSD deviation plot the ACVRIIB-MSTN and MIF1-bound ACVRIIB-MSTN structures attained stability after 4.5 ns, while the MIF2-bound ACVRIIB-MSTN structure attained stability at 6 ns and showed slight fluctuation after 8.5 ns. Overall, all complexes were found to be stable and to exhibit little fluctuation. The radius of gyration (Rg) indicates protein structural compactness as large values indicate structural unfolding. It was found that in all three complexes, Rg fluctuations were between 1.9 and 2.1 nm, where the ACVRIIB-MSTN complex in the presence of MIF1 was found to show greater divergence (between 2.1 and 2.2 nm) (Figure 2B). Overall, Rg values for all the complexes were found to be in range and few fluctuations were noticed, indicating all three complexes maintained compact structures. Thus, MD results showed all three complexes were stable throughout the simulation period.

### 2.2. Myoblast Proliferation and Differentiation with MIF1 and MIF2

MSTN protein effects were observed in C2C12 cells after 2 days of treatment in proliferation or myogenic differentiation media. Cell proliferation and fusion indices were reduced by MSTN protein treatment (Supplementary Figure S1). To check myoblast proliferation, cells were cultured in growth media supplemented with non-modified (MIF1 or MIF2) or modified peptides (MIF1-_NH2_, _Ac_-MIF1, _Ac_-MIF1-_NH2_, MIF2-_NH2_, _Ac_-MIF2, or _Ac_-MIF2-_NH2_) for 1 day. Cell proliferation was increased by MIF1 (11%), _Ac_-MIF1 (24%), MIF2 (6%), or _AC_-MIF2-_NH2_ (33%) versus non-treated controls (Supplementary Figure S2). Therefore, MIF1, _Ac_-MIF1, MIF2, and _Ac_-MIF2-_NH2_ peptides were selected for further studies.

**Figure 3:**
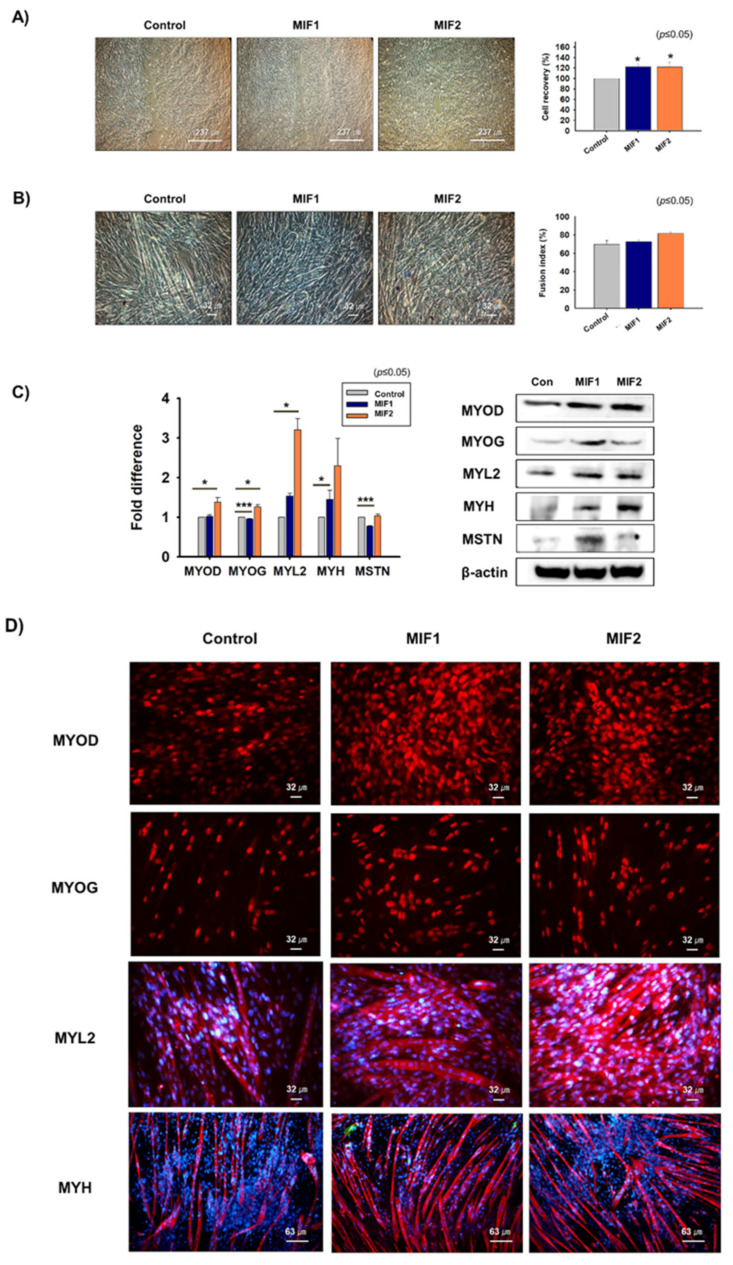
Myoblast proliferation and differentiation in the presence of non-modified MIF 1 and 2 peptides. (**A**) When C2C12 cells reached 100% confluence, cell layers were scratched and incubated with MIF1 or MIF2 for 1 day. Cell proliferation was analyzed using an MTT assay. (**B**–**D**) When cells had reached 70% confluence, growth medium was switched to differentiation medium containing MIF1 or MIF2 and cells were incubated for 3 days. Myotube formation was observed and fusion indices determined by Giemsa staining. mRNA and protein expression of MYOD, MYOG, MYL2, MYH, and MSTN were determined by real-time RT-PCR and Western blot, respectively. Protein localizations were determined by immunocytochemistry. Non-treated cells were used as controls. Means ± SD (n ≤ 3). * *p* ≤ 0.05, ** *p* ≤ 0.001, *** *p* ≤ 0.0001.

Scratch testing was performed on 100% confluent cells, which were incubated in growth medium supplemented with MIF1 or MIF2 for 1 day. Cell recoveries of MIF1- (22%) and MIF2-treated (22%) C2C12 cells were better than those of non-treated cells (Figure 3A). To investigate the effects of MIF1 and MIF2 on myogenic differentiation, 70% confluent cells were switched from growth medium to myogenic differentiation medium supplemented with MIF1 or MIF2 and incubated for 3 days. Myotube formation was increased for MIF1- (4%) or MIF2- (12%) treated cells than for non-treated controls (Figure 3B). Myosin heavy-chain (MYH) mRNA expression was increased and MSTN mRNA expression was decreased in MIF1-treated cells, whereas MSTN mRNA expression in MIF2-treated cells and non-treated controls were similar. MYOD, MYOG, myosin light-chain 2 (MYL2), and MYH proteins expression were increased in MIF1- or MIF2-treated cells, whereas MSTN protein expression increased in MIF1-treated cells but not in MIF2-treated cells (Figure 3C,D). Atrogin1, MuRF1, and ACRVIIB mRNA and protein expression were analyzed in MIF1- and MIF2-treated cells, and ACVRIIB mRNA and protein expression were lower in MIF2-treated cells than in non-treated controls (Supplementary Figure S3). In addition, Smad2 and Smad3 expression were significantly decreased in MIF2-treated cells, while Smad3 expression was decreased in MIF1-treated cells (Supplementary Figure S4). Altogether, these results show that the MIF1 and MF2 peptides enhance myoblast proliferation and differentiation.

### 2.3. Myoblast Proliferation and Differentiation in the Presence of _Ac_-MIF1 or _Ac_-MIF2-_NH2_

**Figure 4:**
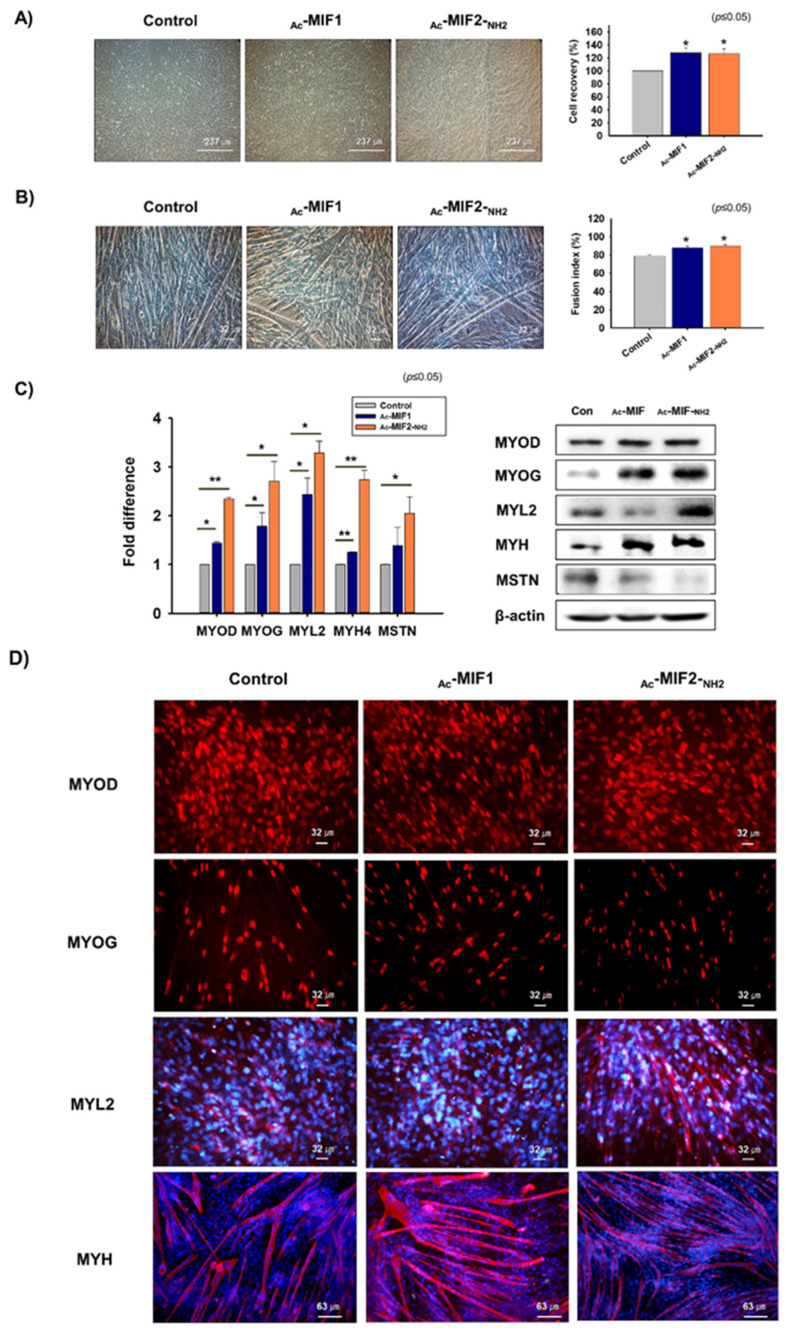
Myoblast proliferation and differentiation with _Ac_-MIF1 & _Ac_-MIF2-_NH2_ peptides. (**A**) When the C2C12 cells reached 100% confluence, layers were scratched and cells were incubated in growth medium supplemented with _Ac_-MIF1 or _Ac_-MIF2-_NH2_ for 1 day. Cell recoveries were analyzed by measuring recovery distances. (**B**–**D**) When cells reached 70% confluence, growth medium was switched to myogenic differentiation medium and cells were incubated with _Ac_-MIF1 or _Ac_-MIF2-_NH2_ for 3 days. Myotube formation was observed and fusion indices determined by Giemsa staining. mRNA and protein expression of MYOD, MYOG, MYL2, MYH, and MSTN were determined by real-time RT-PCR and Western blot, respectively. Protein localizations were determined by immunocytochemistry. Non-treated cells were used as controls. Means ± SD (n ≤ 3). * *p* ≤ 0.05, ** *p* ≤ 0.001, *** *p* ≤ 0.0001.

A Scratch experiment was performed to determine the proliferation effects of _Ac_-MIF1 and _Ac_-MIF2-_NH2_ on C2C12 cells. Cells were incubated in growth media supplemented with _Ac_-MIF1 or _Ac_-MIF2-_NH2_ for 1 day and then cell recoveries were measured. Cell recoveries for _Ac_-MIF1- (28%) and _Ac_-MIF2-_NH2_- (26%) treated cells were better than for non-treated controls (Figure 4A). Mouse primary MSCs were isolated from gastrocnemius muscles and cultured in growth medium supplemented with _Ac_-MIF1 or _Ac_-MIF2-_NH2_ for 1 day. Cell proliferation was significantly greater for _Ac_-MIF1- (9%) or _Ac_-MIF2-_NH2_- (9%) treated cells than for non-treated controls (Supplementary Figure S5A).

C2C12 cells were also cultured in a myogenic differentiation medium supplemented with _Ac_-MIF1 or _Ac_-MIF2-_NH2_ for 3 days. Myotube formation was increased by _Ac_-MIF1 (11%) or _Ac_-MIF2-_NH2_ (14%) (Figure 4B). MYOD, MYOG, MYL2, and MYH mRNA levels and MYOD, MYOG, and MYH protein levels were elevated in _Ac_-MIF1-treated cells, and MYOD, MYOG, MYL2, and MYH mRNA and proteins levels were elevated in _Ac_-MIF2-_NH2_-treated cells. Mouse primary MSCs were cultured with differentiation media supplemented with _Ac_-MIF1 or _Ac_-MIF2-_NH2_ for 3 days. MYOD, MYOG, MYL2, and MYH mRNA expression and MYOD, MYL2, and MYH protein expression were increased in _Ac_-MIF1-treated cells, and MYOD, MYOG, and MYH mRNA and MYOD and MYH protein expression were increased in _Ac_-MIF2-_NH2_-treated cells (Supplementary Figure S5B). Interestingly, MSTN protein levels were reduced in _Ac_-MIF1- and _Ac_-MIF2-_NH2_-treated cells (Figure 4C,D). In addition, Atrogin1 and MuRF1 mRNA levels and ACVRIIB protein levels were lower in _Ac_-MIF1-and _Ac_-MIF2-_NH2_-treated cells than in non-treated controls (Supplementary Figure S6). In addition, Smad3 expression was significantly decreased in _Ac_-MIF1- and _Ac_-MIF2-_NH2_-treated C2C12 cells. However, Smad2 expression was not significantly decreased by the peptide treatment (Supplementary Figure S7). These findings indicate that _Ac_-MIF1 or _Ac_-MIF2-_NH2_ peptides promote myogenesis by increasing the expression of myogenic marker genes.

### 2.4. Effects of MIFs and MSTN Protein on Myogenic Differentiation

**Figure 5:**
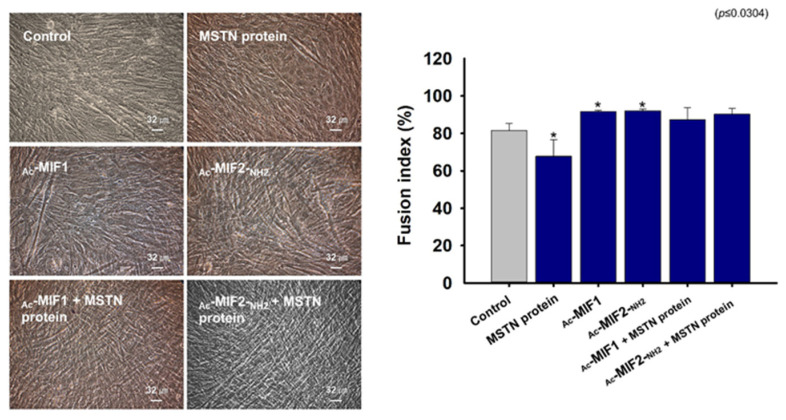
Effects of MSTN protein plus _Ac_-MIF 1 or _Ac_-MIF2-_NH2_ peptide treatments on myogenic differentiation. When C2C12 cells were 100% confluent, growth medium was switched to myogenic differentiation medium supplemented with MSTN protein or _Ac_-MIF1, _Ac_-MIF2-_NH2_, MSTN protein + _Ac_-MIF1, or MSTN protein + _Ac_-MIF2-_NH2_ for 3 days. Myotube formation was observed and fusion indices determined by Giemsa staining. Non-treated cells were used as controls. Means ± SD (n ≤ 3). * *p* ≤ 0.05, ** *p* ≤ 0.001, *** *p* ≤ 0.0001.

C2C12 cells were cultured in growth medium until 70% confluent and the medium was then switched to myogenic differentiation medium supplemented with MSTN-protein, _Ac_-MIF1 or _Ac_-MIF2-_NH2_ for 3 days. Fusion indices were calculated for MSTN-protein-treated, _Ac_-MIF1-treated, _Ac_-MIF2-_NH2_-treated_,_ MSTN-protein + _Ac_-MIF1-treated, and MSTN-protein + _Ac_-MIF2-_NH2_-treated cells. Myotube formation in MSTN-protein-treated cells was lower and _Ac_-MIF1- or _Ac_-MIF2-_NH2_-treated cells were higher than non-treated cells, and myotube formation was greater in MSTN-protein + _Ac_-MIF1-treated or MSTN-protein + _Ac_-MIF2-_NH2_-treated cells than in MSTN-protein-treated cells (Figure 5). These data indicate that _Ac_-MIF1 and _Ac_-MIF2-_NH2_ peptides inhibit the effect of MSTN.

### 2.5. Muscle Regenerative Effects of _Ac_-MIF1 and _Ac_-MIF2-_NH2_

**Figure 6:**
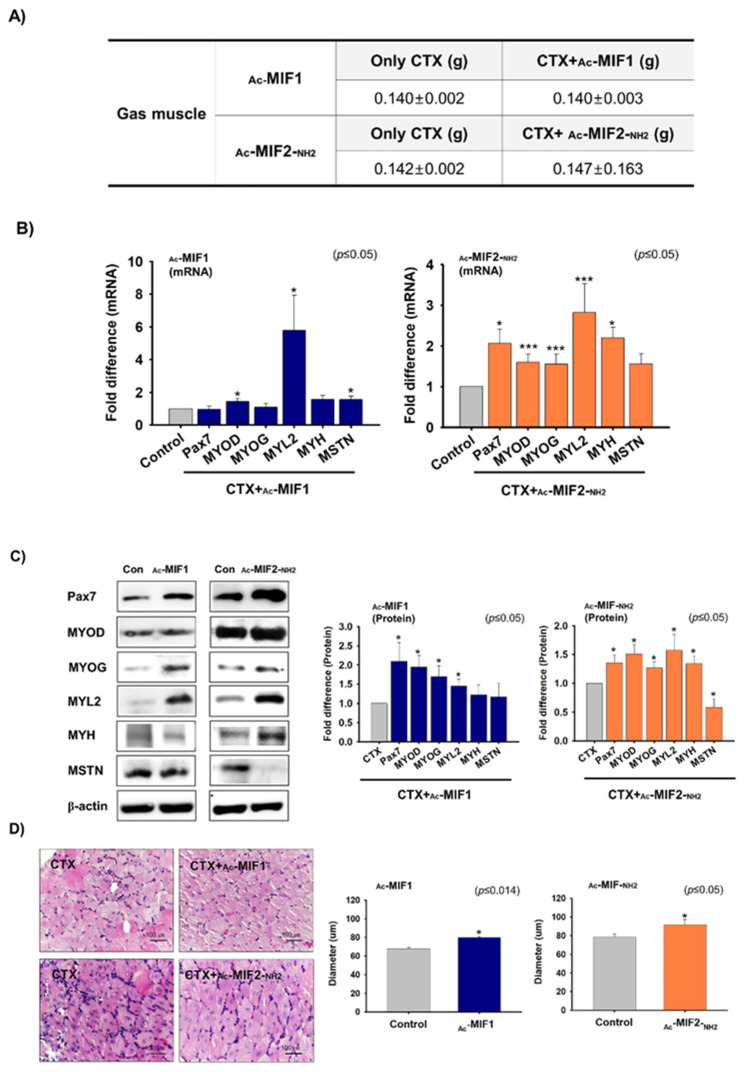
Effects of _Ac_-MIF 1 & _Ac_-MIF2-_NH2_ peptides on muscle regeneration. (**A**) _Ac_-MIF1 or _Ac_-MIF2-_NH2_ peptides were injected into mouse left gastrocnemius muscles. After 1 day, CTX was injected and maintained for 7 days (right leg muscles: CTX injection only, left leg muscles: CTX + _Ac_-MIF1 or _Ac_-MIF2-_NH2_). Body and muscle weights of CTX-injected and CTX + MIF-peptide-injected muscles were measured. (**B**,**C**) Pax7, MYOD, MYOG, MYL2, MYH, and MSTN mRNA and protein expression in CTX-injected and CTX + MIF-peptide-injected muscles as determined by real-time RT-PCR and Western blot, respectively. (**D**) H&E staining and myofiber diameters in CTX-injected and CTX + _Ac_-MIF1-, CTX + _Ac_-MIF2-_NH2_-, and CTX + _Ac_-MIF1 + _Ac_-MIF2-_NH2_-injected muscles. CTX-only-injected muscles were used as controls. Means ± SD (n ≤ 3). (* *p* ≤ 0.05, ** *p* ≤ 0.001, *** *p* ≤ 0.0001).

_Ac_-MIF1 or _Ac_-MIF2-_NH2_ were injected into gastrocnemius muscles and one day later cardiotoxin (CTX) was injected into the left and right muscles for 7 days. Gastrocnemius muscles were then collected and muscle weights (g) were measured for CTX-, CTX + _Ac_- MIF1-, or CTX + _Ac_- MIF2-_NH2_-injected muscles. No significant differences in body or gastrocnemius muscle weights were observed between peptide-injected and non-injected muscles (Figure 6A). However, MYOD, MYL2, and MSTN mRNA expression were higher in _Ac_-MIF1-injected muscles, and Pax7, MYOD, MYOG, MYL2, and MYH mRNA expression were significantly increased in _Ac_-MIF2-_NH2_-injected muscles compared with only CTX-injected muscles (Figure 6B). Pax7, MYOD, MYOG, and MYL2 protein levels were greater in _Ac_-MIF1-injected muscles, while Pax7, MYOD, MYOG, MYL2, and MYH protein levels were greater in _Ac_-MIF2-_NH2_-injected muscles. Interestingly, MSTN protein levels were significantly lower in _Ac_-MIF2-_NH2_-injected muscles (Figure 6C), and ACVRIIB protein levels were lower in _Ac_-MIF2-_NH2_-injected muscles (Supplementary Figure S8). In addition, muscle fiber widths were significantly greater in _Ac_-MIF1-treated muscles than only CTX-injected muscles (Figure 6D). Taken together, _Ac_-MIF1 and _Ac_-MIF2-_NH2_ stimulate muscle regeneration in injured muscles.

### 2.6. Effects of _Ac_-MIF1 and _Ac_-MIF2-_NH2_ on Preadipocyte Proliferation and Differentiation

In previous studies, we showed FMOD regulates MSTN expression by interacting with it and reducing ACVRIIB to MSTN binding affinity, and that lipid accumulation in myoblasts was increased in FMOD knockdown cells [10,18]. In the present study, the gene expression of FMOD and MSTN were analyzed in normal and high-fat diet (HFD) mice adipose tissues. MSTN and FMOD mRNA and protein expression were upregulated and downregulated, respectively, in HFD adipose tissues versus normal adipose tissues (Supplementary Figure S9A). When 3T3L cells reached 100% confluence in the 3T3-L1 growth medium and the medium was switched to adipogenic differentiation media, MSTN and FMOD expression were upregulated and downregulated, respectively, after culture for 4 days (Day 4) as compared with Day 0 (Supplementary Figure S9B). Furthermore, FMOD or MSTN mRNA expression were knocked down in 3T3-L1 cells and cells were cultured in an adipogenic medium. Adipogenic differentiation was observed measuring Oil Red O intensities of control and knockdown cells. Oil Red O intensities were significantly increased in FMOD knockdown cells (FMOD_kd_, 17%) compared with wild-type cells (FMOD_wt_). CD36, PPARγ, and MSTN gene expression were upregulated in FMOD_kd_, while CD36, PPARγ, and FMOD expression were downregulated in MSTN knockdown cells (Supplementary Figures S10 and S11A). In addition, CD36, PPARγ, and FMOD gene expression in MSTN knockout fat tissues were significantly lower than in wild-type fats (controls) (Supplementary Figure S11B).

**Figure 7:**
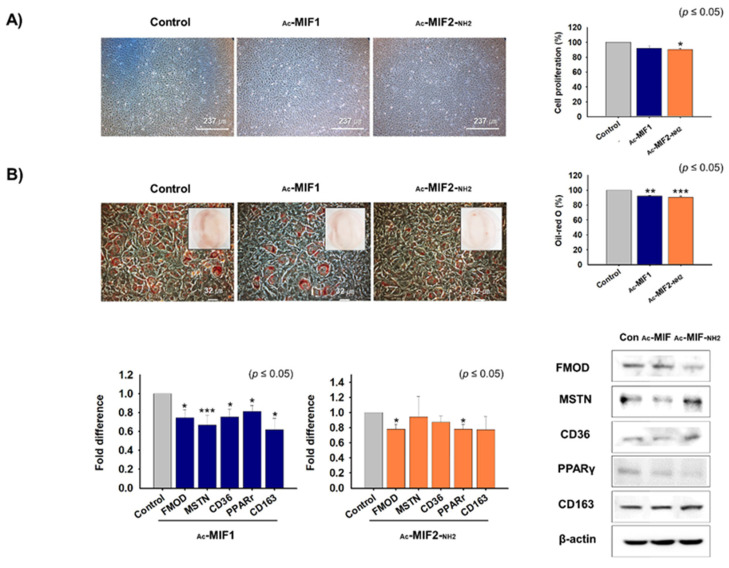
Effects of MIF1-_Ac_ or _Ac_-MIF2-_NH2_ on 3T3-L1 cell proliferation and differentiation. (**A**) 3T3-L1 cells were cultured in 3T3-L1 growth medium supplemented with _Ac_-MIF1 or _Ac_-MIF2-_NH2_ for 2 days. Cell proliferations were analyzed using an MTT assay. (**B**) When cells reached 100% confluence, 3T3-L1 growth medium was switched to adipogenic differentiation medium supplemented with _Ac_-MIF1 or _Ac_-MIF2-_NH2_ peptides for 4 days. Adipogenic differentiation was assessed by Oil Red O staining versus non-treated controls. mRNA and protein expression were determined by real-time RT-PCR and Western blot, respectively. Means ± SD (n ≤ 3). * *p* ≤ 0.05, ** *p* ≤ 0.001, *** *p* ≤ 0.0001.

The effects of MIFs on preadipocyte proliferation and differentiation were investigated in 3T3-L1 cells. First, 3T3-L1 cells were cultured in a 3T3-L1 growth medium supplemented with _Ac_-MIF1 or _Ac_-MIF2-_NH2_ for 2 days. Cell proliferation was significantly suppressed in _Ac_-MIF2-_NH2_-treated cells (10%) versus non-treated cells (controls) (Figure 7A). When cells reached 100% confluence in the growth medium, the medium was switched to adipogenic differentiation medium supplemented with _Ac_-MIF1 or _Ac_-MIF2-_NH2_ for 4 days. Adipogenic differentiation was observed measuring Oil Red O intensities of MIFs-treated and non-treated cells. Adipogenic differentiation was suppressed in _Ac_-MIF1- (8%) or _Ac_-MIF2-_NH2_- (9%) treated cells compared with non-treated cells (control) (Figure 7B). In addition, the mRNA and protein of FMOD, MSTN, and adipogenic markers (CD36, PPARγ, and CD163) were significantly decreased in _Ac_-MIF1-treated cells, and FMOD and PPARγ mRNA and protein expression were decreased in _Ac_-MIF2-_NH2_ (Figure 7B). Furthermore, Smad2 and Smad3 mRNA expression was decreased in _Ac_-MIF-treated cells (Supplementary Figure S12). These results show that _Ac_-MIF1 and _Ac_-MIF2-_NH2_ peptides decrease adipogenesis.

## 3. Discussion

Currently, no approved drug is available for treating muscle-loss-associated diseases such as cachexia and sarcopenia. As these conditions lead to severe morbidity and death, urgent medical intervention is required to address the challenge posed by muscle-related disorders. MSTN is regarded as a therapeutic target for preventing the muscle wasting associated with chronic diseases like cancer. Our study shows that MIF1 and MIF2 peptides not only enhance myogenesis by inhibiting MSTN and inducing myogenic-related markers but that they also reduce adipogenic proliferation and differentiation by suppressing the expression of adipogenic marker genes.

In protein–protein docking, the strength of interaction between two proteins is measured in terms of global energy with a high (negative) global energy value considered as the interaction efficiency of a protein with its receptor [18,30]. In this study, MSTN interacted with ACVRIIB with a global energy of −61.63, while MSTN–MIF1 and MSTN–MIF2 interacted with global energies of −59.69 and −53.91, respectively. These differences between global energies indicate that MIF1 and MIF2 weakened MSTN–ACVRIIB binding. After performing in silico screening, the effects of MIF1 and MIF2 on C2C12 myoblasts were evaluated, and both peptides were found to enhance myoblast proliferation and differentiation by inducing the expression of myogenic marker genes.

Of the different peptide modifications (amidation and acetylation) performed, _Ac_-MIF1 and _Ac_-MIF2-_NH2_ peptides significantly enhanced cell proliferation as compared with non-treated controls and MIF1- or MIF2 peptide-treated cells, and thus _Ac_-MIF1 and _Ac_-MIF2-_NH2_ were subjected to further study. Scratch assays are usually used to compare cell migration parameters such as speed, persistence, and polarity [31]. Interestingly, the scratch assay results of C2C12 myoblasts showed recovery by _Ac_-MIF1- or _Ac_- MIF2-_NH2_-peptide-treated cells was better than for non-treated cells. Furthermore, MSTN protein + _Ac_-MIF1- and MSTN protein + _Ac_-MIF2-_NH2_-treated cells formed more myotubes than MSTN-treated cells, indicating that these peptides suppressed the inhibitory effect of MSTN. Furthermore, in line with these results, _Ac_-MIF1- or _Ac_-MIF2-_NH2_-peptide-treated mouse MSCs also showed enhanced myoblast proliferation.

After confirming the positive effects of _Ac_-MIF1 and _Ac_-MIF2-_NH2_ on myogenesis, we investigated the regenerative potentials of these peptides in CTX-injected mouse gastrocnemius muscles. There were no significant differences in body or gastrocnemius muscle weights between peptide-injected and non-injected muscles, which could be attributed to the fact that the peptide-injected mice experiments were conducted after a short period (7 days). MSCs are responsible for the maintenance and recovery of SM following injury and express nuclear Pax7, which regulates MYOD and MYF5 [32,33]. Furthermore, MYF5, MYOD, MYOG, and MRF4 are crucially involved in directing MSCs to regenerate SM [34]. MSTN sustains the quiescent state of MSCs by negatively regulating Pax7 and its absence results in the proliferation of active MSCs [35,36]. Interestingly, following muscle injury, _Ac_-MIF1 and _Ac_-MIF2-_NH2_ peptides induced muscle regeneration by inducing the protein/mRNA expression of Pax7, MYOD, MYOG, and MYL2. The observed increases in Pax7 and MYOD expression may have been due to increased MSC numbers and subsequent myogenesis due to MSTN inhibition. In addition, reduced ACVRIIB protein expression after _Ac_-MIF2-_NH2_ treatment during muscle regeneration indicated that _Ac_-MIF2-_NH2_ peptide inhibited MSTN by reducing MSTN binding to ACVRIIB. Altogether, these observations suggest _Ac_-MIF1 and _Ac_-MIF2-_NH2_ peptides induce muscle regenerative ability in injured muscles.

In a previous study, inhibition of MSTN by antibody found no change in fat mass in an animal model [37]. However, we found that in addition to enhancing myogenesis, _Ac_-MIF1 and _Ac_-MIF2-_NH2_ both reduced adipogenesis. Other studies have reported adipogenesis was inhibited in 3T3-L1 preadipocytes treated with MSTN during differentiation via the regulations of CCAAT/enhancer-binding protein, PPARγ, and lipid-metabolism-related genes like glycerol-3-phosphate dehydrogenase, diacylglycerol O-acyltransferase, adipose triglyceride lipase, and hormone-sensitive lipase [38,39]. However, here we report that _Ac_-MIF1 and _Ac_-MIF2-_NH2_ peptides suppressed adipogenesis by inhibiting the adipogenic markers CD36, CD163, and PPARγ.

Direct inhibition of MSTN expression has led to positive outcomes in several clinical trials. A randomized, phase 2 clinical trial found that LY2495655 (a humanized MSTN antibody) enhanced appendicular lean mass [40]. Bimagrumab, which was developed by Novartis to treat pathological muscle loss and weakness, is another human monoclonal antibody that targets ACVRIIB receptors. However, several MSTN inhibitors failed to achieve efficacy in clinical trials for the treatment of muscular dystrophy [41,42]. Reports indicate that large-scale, multi-center clinical trials are required to confirm the effectiveness of anti-MSTN therapy.

Peptides are gaining popularity because of their high specificity and biological activities, and because they are relatively inexpensive, which is important as small-molecule drugs are expensive, frequently produce toxic metabolites, and have undesirable side effects. The use of peptides as drugs has progressed considerably and continues to evolve as drugs and treatment paradigms change. Since the advent of insulin over a century ago, peptide therapies have played an important role in medical practice. Existing peptide therapies target a wide range of conditions and are administered intravenously, subcutaneously, via inhalation, and even orally (e.g., linaclotide). In the United States and other major markets, over 80 peptide drugs have been approved for the treatment of a variety of illnesses and conditions such as diabetes, cancer, osteoporosis, multiple sclerosis, HIV, and chronic pain, and therapeutic peptides continue to be developed at an increasing rate [43,44].

MSTN inhibitors offer a new therapeutic approach for various muscle disorders. Age-related sarcopenia and muscle atrophy affect many elderly individuals, and associated reductions in muscle mass and strength seriously diminish the quality of life [45]. Thus, drugs that enhance muscle mass would increase muscle strength and reduce strength-loss-associated injuries like falls and improve quality of life for cachectic patients. In addition, increased SM mass might also have therapeutic benefits for those with muscular dystrophy. Collectively, the present study shows _Ac_-MIF1 and _Ac_-MIF2-_NH2_ peptides both enhance myogenesis, stimulate injured muscle regeneration, and reduce adipogenic proliferation and differentiation by downregulating the expression of adipogenic marker genes. Furthermore, our findings confirm that MSTN inhibition can provide therapeutic benefits for those with debilitating muscular disorders.

In summary, MIFs promote myoblast proliferation, myogenic differentiation, and muscle regeneration by upregulating muscle regulatory genes. On the other hand, they inhibit proliferation and adipogenic differentiation by downregulating adipogenic regulatory genes.

## 4. Materials and Methods

### 4.1. In Silico Analysis

#### 4.1.1. Protein-Protein Interactions (PPIs)

The three-dimensional structures of MSTN (PDB id: 3HH2) and the extracellular domain of activin type IIB receptor (ACVRIIB, PDB id: 1S4Y) were retrieved from the protein databank. I-TASSER webserver was used to model the structure of FMOD using a combined ab initio and threading approach (http://zhanglab.ccmb.med.umich.edu/I-TASSER/ (accessed on 14 March 2021)). PPI studies of interactions between MSTN (complexed with FMOD or not) and ACVRIIB were performed using PatchDock (https://bioinfo3d.cs.tau.ac.il/PatchDock/ (accessed on 21 March 2021)) and further refined and ranked using FireDock (http://bioinfo3d.cs.tau.ac.il/FireDock/ (accessed on 21 March 2021)) web servers [46]. PatchDock generated 100 predictions for each interaction, which were then processed using FireDock to generate the 10 best solutions based on global energy.

#### 4.1.2. Binding Pattern Analysis

A series of evaluations was performed to identify common binding patterns. MSTN interactions with FMOD and ACVRIIB were analyzed and the MSTN residues that participated most were selected. Pattern studies were performed using various approaches such as changes in accessible surface area (ASA) and virtual alanine scanning (available at http://robetta.bakerlab.org/alaninescan (accessed on 25 March 2021)) to identify residues that contribute most to complex formation.

#### 4.1.3. Peptide Screening

The inhibitory efficacies of designed peptides against MSTN were predicted using an in silico binding approach. All designed peptides were docked with MSTN, and binding studies were performed using Patchdock followed by FireDock [46]. The top-scoring peptides were selected based on their global binding energies with MSTN.

#### 4.1.4. Molecular Dynamics (MD)

An available crystal structure of MSTN complexed with follistatin288 was used as a starting point for the conformational dynamics study. The GROMACS 4.6.7 package [47] was used for system preparation and MD simulations were run using the gromos96 force field [48]. The explicit SPC water model was used to solvate protein in a dodecahedron box by taking a margin of 0.1 nm from solute to simulation box. The system was neutralized by adding Na^+^ and Cl^−^ ions to a concentration of 0.1 M. Following solvation and neutralization, the system was energetically reduced over 10,000 steps using the steepest-descent approach to eliminate steric clashes between atoms. The equilibration process was divided into two stages: NVT ensemble and NPT ensemble. Original structures of backbone atoms were restrained, whereas all other atoms were free to move in both NVT and NPT ensembles. Further, production runs were performed in the isothermal-isobaric (NPT) ensemble for 10 ns without constraints at 300 K. For the analysis, several software packages were used such as xmgrace (http://plasmagate.weizmann.ac.il), VMD [49], and PyMol (The PyMOL Molecular Graphics System, Version 1.7 Schrödinger, LLC., New York, NY, USA).

### 4.2. MIF Peptide Synthesis

The eight MIF peptides selected by in silico studies were named MIF1, MIF2, _Ac_-MIF1, _Ac_-MIF2, MIF1-_NH2_, MIF2-_NH2_, _Ac_-MIF1-_NH2_, and _Ac_-MIF2-_NH2_, synthesized by Peptron (Daejeon, Korea), diluted with DMSO (Sigma Aldrich, St. Louis, MO, USA), and stored at −20 °C.

### 4.3. Animal Experiments

C57BL/6 male mice (6–9 weeks old) were purchased from Daehan Biolink (Daejeon, South Korea) and kept four per cage in a temperature-controlled room (normal diet containing 4.0% (*w*/*w*) total fat or high-fat diet (HFD) containing 45% fat). All animal-related experiments followed the guidelines issued by the Institutional Animal Care and Use Committee of Catholic University of Daegu and Yeungnam University (IACUC-2014-035, YUMC-AEC2018-031).

To investigate the effects of _Ac_-MIF1 and _Ac_-MIF2-_NH2_ on muscle regeneration, 80 µL of 1.125 mM _Ac_-MIF1 or _Ac_-MIF2-_NH2_ were injected into left gastrocnemius muscles. Contralateral gastrocnemius muscles were injected with phosphate buffer saline (PBS) and used as controls. One day later, 100 µL of 100 nM cardiotoxin (CTX; Sigma Aldrich) was injected into left and right gastrocnemius muscles and incubated for 7 days. CTX/non-peptide-injected and CTX- _Ac_-MIF1- or _Ac_-MIF2-_NH2_-injected muscle tissues were collected for protein extraction or fixed for H&E (hematoxylin and eosin) staining. MSNT knockout mice [50] were provided by Lee’s lab at Seoul National University. Normal and MSTN knockout (MSTN^−/−^: homozygote) epididymis fat tissues were collected from 6 weeks old and stored at −80 °C for protein analysis. All treatments were administered to anesthetized (Avertin i.p.) animals.

### 4.4. Mouse MSC Isolation

Mouse MSCs were isolated and cultured using our previously described protocol [51].

### 4.5. C2C12 Cell Culture

C2C12 cells (Korean Cell Line Bank, Seoul, Korea) were grown in growth medium (DMEM (HyClone Laboratories, South Logan, UT, USA) + 10% FBS + 1% P/S) at 37 °C in a 5% CO_2_ atmosphere. The medium was changed every other day.

### 4.6. C2C12 Cell Differentiation

C2C12 cells were grown in growth medium until 70% confluent, and then in myogenic differentiation medium (DMEM + 2% FBS + 1% P/S) supplemented with MIF1, MIF2, _Ac_-MIF1 and _Ac_-MIF2-_NH2_ for 3 days to promote myoblast differentiation. Media were changed daily.

### 4.7. 3T3-L1 Cell Culture

Mouse embryonic fibroblasts (3T3-L1 cells; Korean Cell Line Bank, Seoul, Korea) were grown in 3T3-L1 growth medium (DMEM + 10% Newborn calf serum (HyClone Laboratories) + 1% P/S) at 37 °C in a 5% CO_2_ atmosphere. The medium was changed every other day.

### 4.8. 3T3-L1 Cell Differentiation

3T3-L1 cells were grown until 100% confluent, in adipogenic differentiation medium (DMEM + 10%FBS + 1%P/S + 10 µg/mL insulin (Sigma-Aldrich, St. Louis, MO, USA) + 1 µM dexamethasone (Sigma-Aldrich, St. Louis, MO, USA) + 0.5 µM 3-isobutyl-1-methylxanthine (IBMX, Sigma Aldrich, St. Louis, MO, USA)) supplemented with _Ac_-MIF1 and _Ac_-MIF2-_NH2_ for 2 days, and then in 3T3-L1 growth media supplemented with 10 µg/mL insulin and _Ac_-MIF1 or _Ac_-MIF2-_NH2_ for another 2 days.

### 4.9. Gene Knockdown

3T3-L1 cells were transfected with FMOD, MSTN shRNA, and scrabbled vector. Knockdown and selection was performed as we previously described [50].

### 4.10. MSTN Protein Treatment

C2C12 cells were cultured in growth or myogenic differentiation medium supplemented with 0.5 or 1 ng of MSTN proteins (Invitrogen, Carlsbad, CA, USA), respectively, for 2 days. When cells reached 70% confluence, growth media was switched to myogenic differentiation media supplemented with MSTN proteins (1 ng), _Ac_-MIF1 (1000 nM), _Ac_-MIF2-_NH2_ (1000 nM), MSTN protein (1 ng) + _Ac_-MIF1 (1000 nM), or MSTN protein (1 ng) + _Ac_-MIF2-_NH2_ (1000 nM) for 3 days.

### 4.11. MTT Assay

C2C12 cells, 3T3-L1 cells, or mouse MSCs (1000 cells/mL) were cultured in growth media supplemented with 1000 nM MIF peptides (MIF1, MIF2, _Ac_-MIF1, _Ac_-MIF2, MIF1-_NH2_, MIF2-_NH2_, _Ac_-MIF1-_NH2_, or _Ac_-MIF2-_NH2_) for 1 day. Media was then removed, and cells were washed with DMEM (C2C12 and 3T3-L1 cells) or Ham’s F10 (Mouse MSCs) media and then incubated with 0.5 mg/mL of MTT reagent (Sigma-Aldrich, St. Louis, MO, USA) for 1 h at 37 °C. The formazan crystals formed were dissolved in DMSO, and absorbance was measured at 540 nm using a Versa Max microplate reader (Tecan Group Ltd., Männedorf, Switzerland).

### 4.12. Giemsa Staining and Fusion Indices

When C2C12 cells reached 70% confluence, growth medium was switched to myogenic differentiation medium supplemented with MIF1, MIF2, _Ac-_MIF1, or _Ac-_MIF2-_NH2_ for 3 days. Giemsa staining was performed and fusion indices were determined as we previously described [52].

### 4.13. Oil Red O Staining

3T3-L1 cells in adipogenic medium supplemented with _Ac_-MIF1 or _Ac_-MIF2-_NH2_ were cultured for 4 days and washed with PBS, fixed with 10% formaldehyde (Sigma-Aldrich, St. Louis, MO, USA) for 10 min, and incubated in Oil Red O solution (6:4 dilution of stock (3.5 mg/mL Oil Red O powder in 100% isopropanol)) for 1 h and washed with PBS. Stained cells were examined under a microscope and photographed using a digital camera (Nikon, Tokyo, Japan). To quantify intracellular Oil Red O levels, 100% isopropanol (Merk KGaA, Darmstadt, Germany) was added, collected, and absorbance was measured at 510 nm using Versa Max microplate reader.

### 4.14. Real-Time RT-PCR

Total RNAs from cells muscle and adipose tissues were extracted using Trizol reagent (Thermo Fisher Scientific, Waltham, MA, USA). Real-time RT-PCR was performed as we previously described [52]. Primer information is provided in Supplementary Table S6.

### 4.15. Western Blot

Total proteins of C2C12, 3T3-L1 cells, muscle, or fat tissues were extracted using RIPA buffer supplemented with protease inhibitor (Thermo Fisher Scientific, Waltham, MA, USA), and protein concentrations were measured using the Bradford assay. For western blotting, proteins (40 or 60 µg) were electrophoresed in 6, 10, or 12% SDS-polyacrylamide gels and transferred to PVDF membranes (EMS-Millipore, Billerica, MA, USA). Membranes were then incubated in blocking reagent (3% skim milk or BSA in Tris-buffered saline (TBS)/Tween 20) and treated overnight with specific primary antibodies (FMOD (1:400), MSTN (1:1000), Pax7 (1:500), MYOD (1:500), MYOG (1:500), MYH (1:500), MuRF1 (1:500), Atrogin1 (1:500), or β-actin (1:1000) antibodies (Santa Cruz Biotechnology), or MYL2 (1:1000, Abcam, Cambridge, MA, USA), ACVRIIB (1:500, Abcam)) in 1% skim milk or BSA in TBS at 4 °C. After washing, membranes were incubated with goat-rabbit or mouse-horseradish-peroxidase (HRP)-conjugated secondary antibodies (Santa Cruz Biotechnology) for 1 h at room temperature. Blots were developed using Super Signal West Pico Chemiluminescent Substrate (Thermo Fisher Scientific, Waltham, MA, USA).

### 4.16. Immunocytochemistry

After removing media, cells were washed with PBS, fixed with 4% formaldehyde (Sigma Aldrich) for 15 min, incubated with 0.2% Triton X 100 (Sigma Aldrich) for 5 min, washed with PBS, incubated with 1% normal goat serum (SeraCare Life Sciences, Milford, MA, USA) for 30 min in a humid environment, and then incubated with protein-specific primary antibodies (MYOD (1:50), MYOG (1:50), MYL2 (1:50), or MYH (1:50)) at 4 °C in a humid environment overnight. Alexa Fluor 594 goat anti-rabbit or anti-mouse secondary antibodies (1:100; Thermo Fisher Scientific, Waltham, MA, USA) were then added, left for 1 h at room temperature, and cells were stained with DAPI (Sigma-Aldrich, St. Louis, MO, USA). Images were taken using a fluorescence microscope equipped with a digital camera (Nikon, Japan).

### 4.17. H&E Staining and Muscle-Fiber Diameter Measurements

Paraffin-embedded muscle sections were deparaffinized with xylene (Junsei, Tokyo, Japan), rehydrated using an ethanol gradient, stained with H&E (Thermo Fisher Scientific), and examined under a light microscope (Leica, Wetzlar, Germany). Muscle diameters were measured using Image J software [53].

### 4.18. Immunohistochemistry

Immunohistochemistry was performed as previously described [52]. Briefly, muscle tissue sections were deparaffinzed, hydrated, quenched, and blocked with 1% normal goat serum, then incubated with ACVRIIB antibody (1:50) overnight at 4 °C and treated with HRP-conjugated antibody (1:100, Santa Cruz Biotechnology, CA, USA) at room temperature for 1 h. Tissue sections were counterstained with hematoxylin, dehydrated, mounted, and observed under a light microscope (Leica).

### 4.19. Statistical Analysis

Tukey’s Studentized Range (HSD) test and the *t* test were used to determine the significance of the differences between mean normalized gene expressions. GAPDH or β-actin were used as internal controls, and the analysis was carried out by one-way ANOVA using PROC GLM in SAS ver. 9.0 (SAS Institute, Cary, NC, USA). Statistical significance was accepted for *p* values < 0.05.

## Figures and Tables

**Table 1:** **List of the MSTN peptides examined.** The table shows the peptide sequences, sizes, molecular formulae, and molecular weights of non-modified and modified (by acetylation and/or amidation) peptides.

	Peptide	Sequence	Mer	Molecular Formula	M.W
Non-modified	MSNT1	VDFEAFWDWG	10	C_63_H_74_N_12_O_17_	1270.53
	MSTN2	VDFEAGDWFW	10	C_63_H_74_N_12_O_17_	1270.53
Modified	_Ac_-MSTN1	_Ac_-VDFEAFWDWG	10	C_65_H_76_N_12_O_18_	1312.54
	_Ac_-MSTN2-_NH2_	_Ac_-VDFEAGDWFW-_NH2_	10	C_65_H_77_N_13_O_17_	1311.56

## Data Availability

The data presented in this study are available in this article and the accompanying Supplementary Materials.

## Supplementary Materials

The following supporting information can be downloaded at: https://www.mdpi.com/article/10.3390/ijms23084222/s1.
