# Supplementary material for: MIF1 and MIF2 Myostatin Peptide Inhibitors as Potent Muscle Mass Regulators
Source: Int J Mol Sci. 2022 Apr 11;23(8):4222. doi: 10.3390/ijms23084222 (PMC9031736; doi:10.3390/ijms23084222)

Supplementary Table S1.

MSTN	ASA	BSA
**VAL 22**	22.56	17.54
**ASP 23**	37.06	0.00
**PHE 24**	4.90	1.72
**GLU 25**	109.71	0.00
**ALA 26**	88.43	0.00
**PHE 27**	120.38	60.67
**GLY 28**	42.06	6.04
**TRP 29**	104.89	91.03
**ASP 30**	126.32	0.28
**TRP 31**	113.64	69.08

Supplementary Table S2.

MSTN	ASA	BSA
**VAL 22**	**21.60**	**18.09**
**ASP 23**	37.17	0.00
**PHE 24**	5.04	1.41
**GLU 25**	109.77	2.95
**ALA 26**	87.63	34.72
**PHE 27**	122.64	104.82
**GLY 28**	42.85	26.64
**TRP 29**	104.93	98.26
**ASP 30**	124.60	0.00
**TRP 31**	116.87	24.46

Supplementary Table S3.

MSTN	ASA	BSA
**VAL 22**	22.43	17.74
**ASP 23**	37.12	0.00
**PHE 24**	5.34	2.18
**GLU 25**	109.51	0.00
**ALA 26**	87.16	0.00
**PHE 27**	124.28	60.98
**GLY 28**	42.32	6.05
**TRP 29**	103.30	89.84
**ASP 30**	124.36	0.28
**TRP 31**	114.43	69.56

Supplementary Table S4.

Residue	DDG (complex)	DDG (complex, obs)	DG (partner)
22	0.37	0	1.86
27	2.08	0	0.59
29	2.13	0	1.33
31	-0.11	0	1.52
32	-0.27	0	3.12
38	0.13	0	4.75
42	0.81	0	1.88
55	0.10	0	0.02
73	-0.05	0	5.78
75	1.29	0	0.58
79	-2.31	0	1.40
84	-0.17	0	2.05
98	0.36	0	1.51
101	0.39	0	1.82
103	0.00	0	2.22
109	0.10	0	-0.49
22	0.37	0	1.86

Supplementary Table S5.

Residue	DDG (complex)	DDG (complex, obs)	DG (partner)
20	0.33	0	1.23
22	0.38	0	1.86
24	0.31	0	3.21
27	0.99	0	0.59
29	4.15	0	1.33
31	0.36	0	1.52
38	0.25	0	4.75
42	0.63	0	1.89
43	-0.03	0	5.17
44	-0.07	0	-0.45
55	1.59	0	0.02
57	1.1	0	-1.06
58	0.00	0	0.52
60	-0.02	0	0.61
61	0.65	0	0.59
67	0.19	0	-0.58
69	-0.11	0	0.58
73	-0.37	0	5.78
74	-0.01	0	5.26
75	0.14	0	0.57
79	0.14	0	1.40

88	-0.01	0	3.27
98	-0.01	0	1.51
101	0.35	0	1.82
103	0.09	0	2.22
109	-0.06	0	-0.48

Supplementary Table S6.

Species	Gene	Product size (bp)	Tm (°C)	Sequence (F)	Sequence (R)
Mouse	GAPDH	155	59	5'-tgctggtgctgagtatgtcg-3'	5'-caagcagttggtggtacagg-3'
Mouse	Pax7	170	59	5'-gagttcgattagccgagtgc-3'	5'-cgggttctgattccacatct-3'
Mouse	MYOD	213	59	5'-aggagcacgcacacttctct-3'	5'-tctcgaaggcctcattcact-3'
Mouse	MYOG	185	59	5'-tccagtacattgagcgccta-3'	5'-caaatgatctcctgggttgg-3'
Mouse	MYL2	177	59	5'-aaagaggctccaggtccaat-3'	5'-cctctctgcttgtgtggtca-3'
Mouse	MYH	248	59	5'gggttccattgacattgacc-3	5'-agggccagtgtttcacattc-3'
Mouse	Atrogin1	160	59	5'-ttcagcagcctgaactacga-3'	5'-tgaaagcttcccccaaagta-3'
Mouse	MuRF1	206	59	5'-tgaggtgcctacttgctcct-3'	5'-tcacctggtggctattctcc-3'
Mouse	MSTN	163	59	5'-acgctaccacggaaacaatc-3'	5'-ggagtcttgacgggtctgag-3'
Mouse	ACVRIIB	197	59	5'aacttccagagagacgcctt-3'	5'atcgtgggcctcatcttctt-3'
Mouse	CD36	187	59	5'-tggagctgttattggtgcag-3'	5'-tgggttttgcacatcaaaga-3'
Mouse	PPARγ	232	59	5'-aagagctgacccaatggttg-3'	5'-accttgcatccttcacaag-3'
Mouse	CD163	218	59	5'-ctggtcgtgtggaagtgaaa-3'	5'-cgccactgagcatagtgaaa-3'
Mouse	Smad 2	209	59	5'-gagctcaaggcaatcgaaaa-3'	5'-cctgctgggaaatttgtgtt-3'
Mouse	Smad 3	197	59	5'-ctgggcctactgtccaatgt-3'	5'-ggtgggatcttgcagacagt-3'

Supplementary Figure S1.

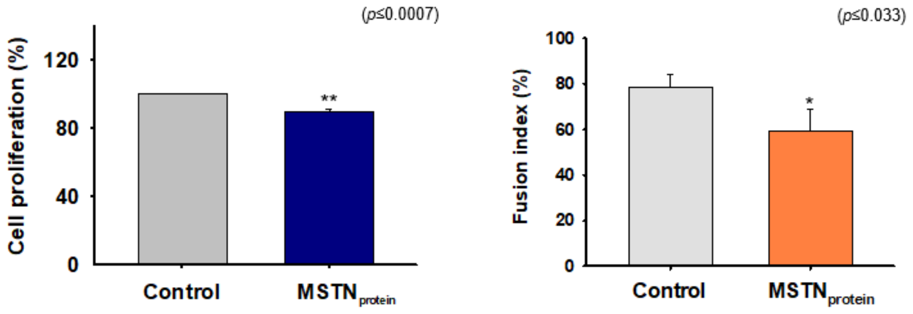

Supplementary Figure S2.

	Peptide	Sequence
Non-modification	MSNT1	VDFEAFWDWG
	MSTN2	VDFEAGDWFW
Modification	MSTN1-$_{NH2}$	VDFEAFWDWG-$_{NH2}$
	$_{Ac}$-MSTN1	$_{Ac}$-VDFEAFWDWG
	$_{Ac}$-MSTN1-$_{NH2}$	$_{Ac}$-VDFEAFWDWG-$_{NH2}$
	MSTN2-$_{NH2}$	VDFEAGDWFW-$_{NH2}$
	$_{Ac}$-MSTN2	$_{Ac}$-VDFEAGDWFW
	$_{Ac}$-MSTN2-$_{NH2}$	$_{Ac}$-VDFEAGDWFW-$_{NH2}$

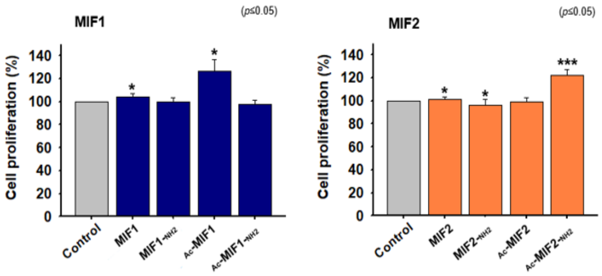

Supplementary Figure S3.

Gene	MIF1	MIF2
Atrogin1	0.74 ± 0.07*	0.74 ± 0.19
MuRF1	1.20 ± 0.32	1.14 ± 0.28
ACVRIIB	0.76± 0.14	0.48 ± 0.08*

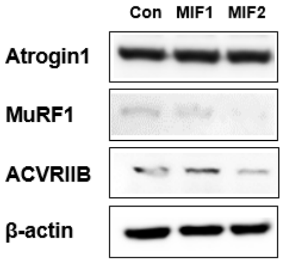

Supplementary Figure S4.

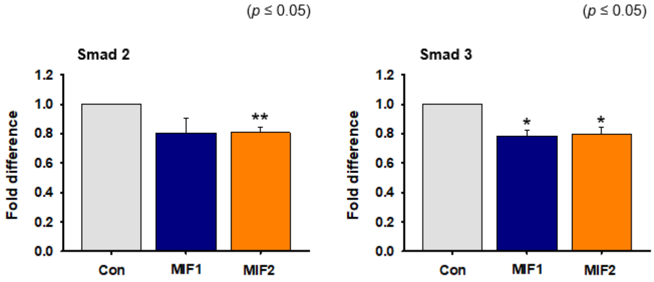

Supplementary Figure S5.

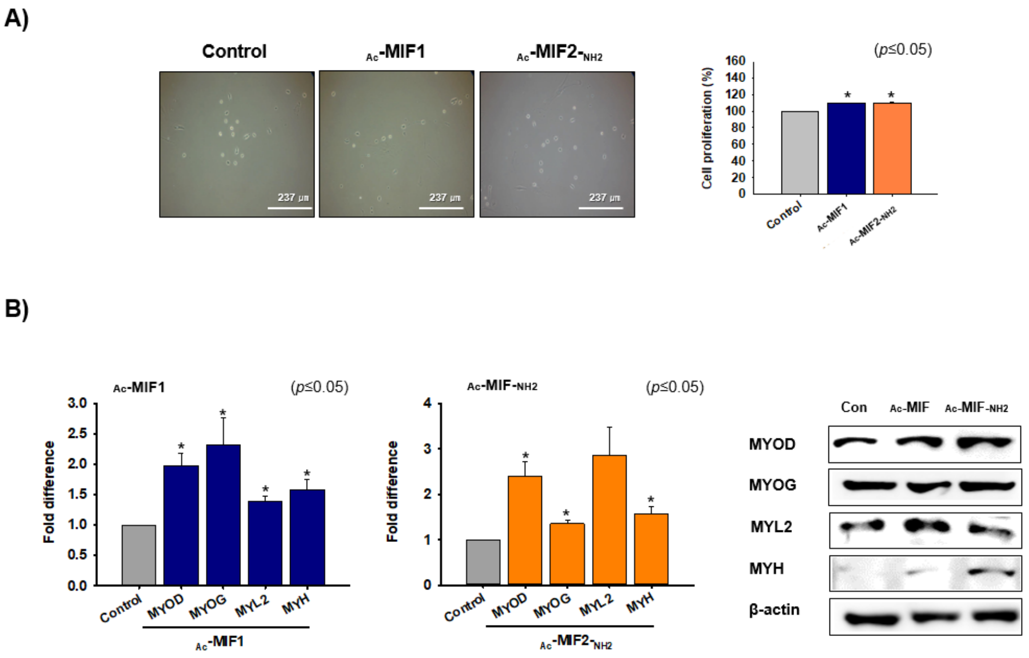

Supplementary Figure S6.

Gene	$_{Ac}$-MIF1	$_{Ac}$-MIF2-$_{NH2}$
Atrogin1	0.91 ± 0.07	0.90 ± 0.02*
MuRF1	0.64 ± 0.16	0.92 ± 0.04
ACVRIIB	0.87 ± 0.17	0.79 ± 0.15

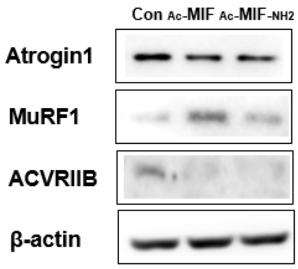

Supplementary Figure S7.

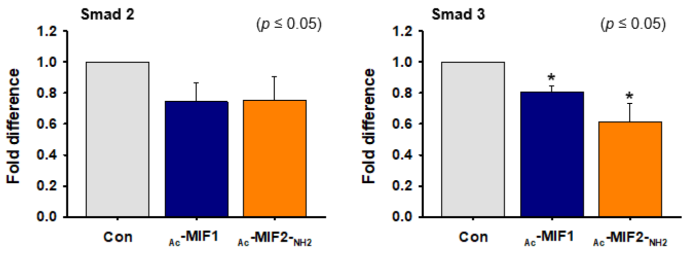

Supplementary Figure S8.

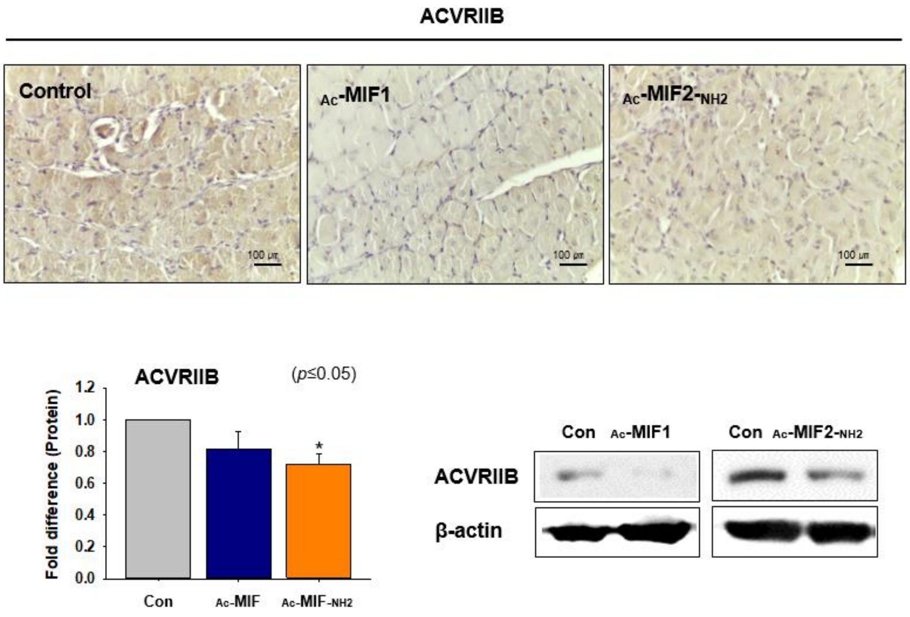

Supplementary Figure S9.

A)

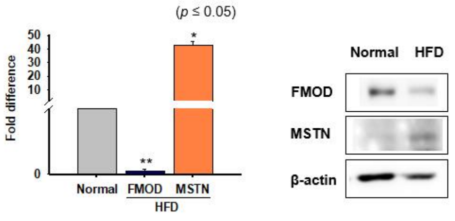

B)

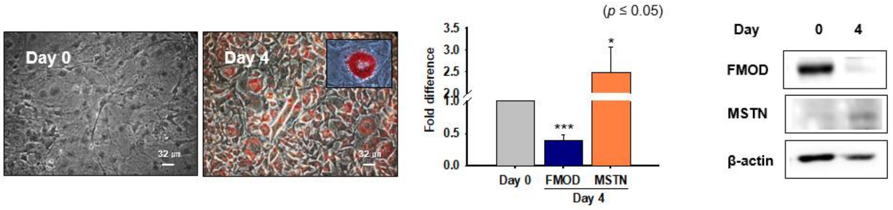

Supplementary Figure S10.

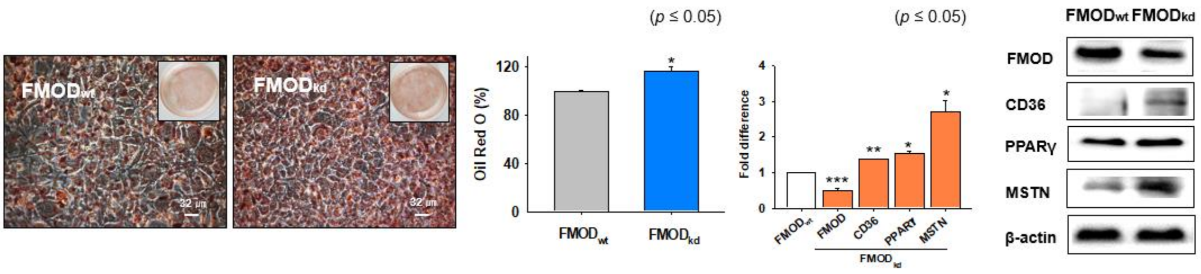

Supplementary Figure S11.

A)

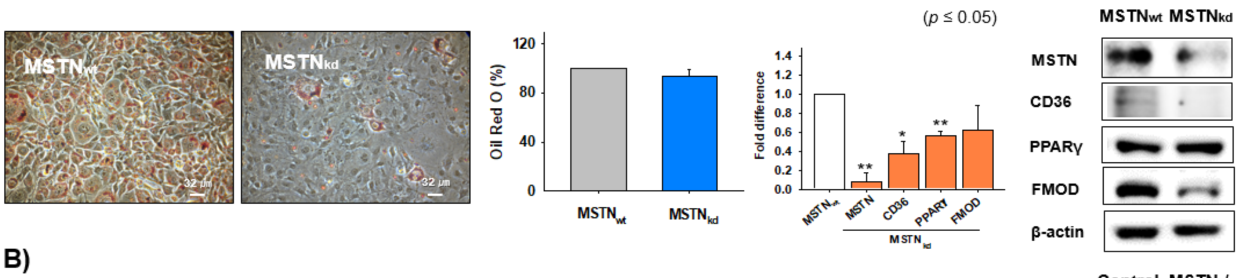

B)

Gene	Fold difference	Significance
CD36	0.13±0.031	***
PPARγ	0.05±0.011	***
FMOD	0.01±0.003	***

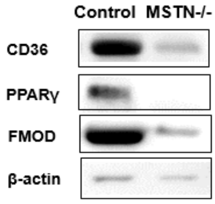

Supplementary Figure S12.

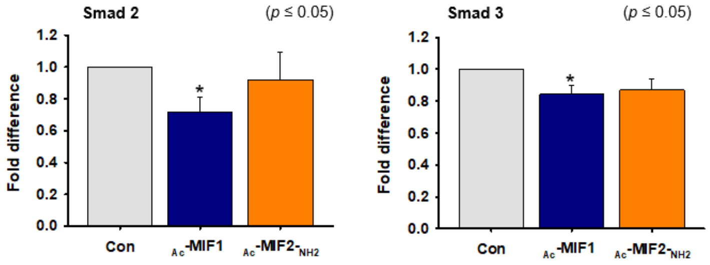

Supplement: Supplementary file 1 [file ijms-23-04222-s001.zip › ijms-1657995-supplementary.pdf]
